# Hereditary Hemorrhagic Telangiectasia Presenting as High Output Cardiac Failure during Pregnancy

**DOI:** 10.4061/2009/437237

**Published:** 2009-09-07

**Authors:** Tareq Goussous, Alex Haynes, Katherine Najarian, Marcos Daccarett, Shukri David

**Affiliations:** ^1^Division of Cardiology, Providence Heart Institute, Southfield, MI 48075, USA; ^2^School of Medicine, Wayne State University, Detroit, MI 48201, USA

## Abstract

High-output cardiac failure secondary to hepatic involvement is a rare complication of hereditary hemorrhagic telangiectasia (HHT). Here we report a 43-year-old woman who presented at 29 weeks gestation of her second pregnancy with complications of right-sided heart failure and preterm labor. After delivery via cesarean section, the patient was found to have intrahepatic arteriovenous malformations through non-invasive imaging. Subsequently, a family history of vascular malformations and epistaxis was elucidated and a diagnosis of HHT was made. This case is presented, along with a review of the literature and discussion of hepatic involvement in HHT with particular focus on the pregnant patient.

Hereditary hemorrhagic telangiectasia (HHT), also known as Osler-Weber-Rendu disease, is an autosomal dominant disorder affecting vascular structures. The prevalence of the disorder has been found to be from 1 in 2,351 to 1 in 39,216 varying by geography. Its hallmark is telangiectatic lesions, most frequently involving the skin and mucous membranes. Visceral involvement is common, with the lungs, brain, alimentary tract, and liver as the most common sites of arteriovenous malformations. Patients may present with epistaxis, cutaneous lesions, gastrointestinal bleeding, or other symptoms [1].

A 43-year-old G2P2 presented on postpartum day 2 with complaints of severe orthopnea, nocturnal paroxysmal dyspnea, and lower extremity edema. These symptoms had begun during the second trimester of pregnancy and had gradually progressed. Her medical history is positive for recurrent epistaxis, chronic anemia, and mitral valve regurgitation. The patient's mother had a history of recurrent epistaxis and had experienced pregnancy complications that were similar to the current symptoms of the index patient. On review of the mother's medical records, it was found that she had been diagnosed with arteriovenous malformations in her liver through ultrasonography. The maternal aunt was also reported to have similar complains.

On examination, she had a holosystolic murmur of II/VI intensity that was audible throughout the precordium, which was markedly hyperdynamic. Her abdomen was distended and her liver was palpable six centimeters below the costal margin. There was a continuous bruit audible over the liver and she demonstrated hepatojugular reflux. An echocardiogram demonstrated normal left ventricular function with an ejection fraction of 60% with moderate mitral regurgitation and mild tricuspid regurgitation with structurally normal valves. There was no evidence of ventricular hypertrophy or enlargement.

Her laboratory findings were significant for a normochromic, normocytic anemia, decreased albumin (2.8 g/dL) and total protein (4.8 g/dL). She had mildly elevated total bilirubin (1.8 mg/dL), aspartate transaminase and alanine transaminase.

Computerized tomography (CT) and magnetic resonance imaging (MRI) of the abdomen and pelvis performed demonstrated an enlarged, nodular, heterogeneous liver along with ascites. Two lesions were observed in the lungs that were consistent with arteriovenous malformations (AVMs) (Figure 1). An abdominal ultrasound revealed the hepatic nodularity to be due to hypervascularity. Spectral Doppler evaluation of these vessels revealed them to have mixed arterial and venous waveforms, consistent with multiple arteriovenous malformations of the liver. No enlargement of the spleen was observed.

Marked symptomatic improvement was demonstrated after heart failure management with metoprolol, furosemide, and spironolactone. She was discharged from the hospital on postpartum day 14 with her abdominal pain relieved and lower extremity edema resolving.

The diagnosis of HHT is made through clinical findings. Patients have a combination of recurrent epistaxis, mucocutaneous telangiectases, visceral arteriovenous malformations, and affected first-degree family members. The disease is considered to be present if 3 of these 4 criteria are met and possible if 2 are present [2]. Radiological adjuncts to diagnosis include sonography, angiography, CT, and MRI. The ability to delineate flow through Doppler modalities makes ultrasonography an appealing technology for diagnosis of visceral disease and recent efforts have been made to describe parameters for diagnosis.

It has recently been determined that HHT is a cluster of autosomally dominant disorders that are the result of various genetic defects. Specific genes in which mutations have been identified include endoglin and ALK-1, which are responsible for subtypes HHT-1 and HHT-2, respectively. It is thought that the differing mutations may be partially responsible for the heterogeneity of the disease although this has not been fully characterized [3].

The liver manifestations found in HHT include high output cardiac failure, portal hypertension, and biliary disease [4]. Usually the cardiovascular manifestations prevail, with only mild liver impairment. However, cases have been described of liver failure due to intrahepatic HHT lesions [5]. Hepatic involvement in HHT is present in a significant portion of patients with the disease, with estimates of frequency ranging from 8% to 31% [6]. A meta-analysis of families reported to have HHT-2 found evidence of intrahepatic shunting in 13% of the 281 patients [7]. However, since hepatic lesions may often be asymptomatic, it is difficult to accurately assess the prevalence and significance of these AVMs. One study found hepatic involvement in 13 of 40 family members who were screened with ultrasound, but only 2 of these individuals had symptoms attributable to the lesions [8].

Vascular lesions in the liver can include either hepatic artery-hepatic vein or hepatic artery-portal vein malformations. When the anomalous vasculature involves the portal vein, portal hypertension and the related symptoms may evolve. The patient may present with ascites, bleeding varices, and rarely encephalopathy. More commonly, the disease affects the hepatic artery and vein. First described in 1971, high-output cardiac failure is the most common manifestation of large hepatic artery to hepatic vein shunting [9]. Reported cases of right-heart catheterization in patients with symptomatic hepatic AVMs have calculated shunts of 24% to 58% of the cardiac output.

Only 26 cases of symptomatic visceral HHT during pregnancy have been reported. The majority of these cases involve pulmonary lesions, with nine reported cases of hepatic involvement during pregnancy. The cardiovascular changes of pregnancy-increased intravascular volume and venous distensibility, as well as increased cardiac output, may contribute to the enlargement of arteriovenous malformations during this period. These changes resolve over weeks to months postpartum. This may explain the spontaneous resolution of symptoms observed in some of these patients after delivery [10].

Therapeutic options for patients with peripartum high-output cardiac failure secondary to HHT remain controversial. There is evidence that some patients may spontaneously get better after delivery while in other patients the disease is progressive. Consequently, a period of conservative management is warranted in stable patients before considering invasive therapy. Medical therapy can help optimize cardiac function and alleviate symptoms. There has been enthusiasm for transcatheter embolization of hepatic AVMs in the past [11]. This technique has been reported to be successful in patients with pulmonary lesions. However, there have been reports of frequent morbidity and mortality of the procedure when applied to hepatic AVMs [12]. Alternatively, liver transplantation has been proposed as a treatment for intractable right heart failure or in the case of overt liver dysfunction [13]. This therapy has been effective in resolving high-output heart failure, although it has its own attendant mortality and morbidity.

## Figures and Tables

**Figure 1:**
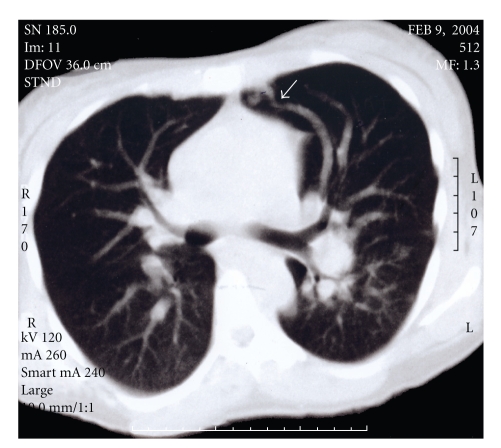
Axial computerized tomography of the chest in lung window demonstrating a feeding artery and a draining vein to a nidus of an arteriovenous malformation (arrowhead).

**Figure 2:**
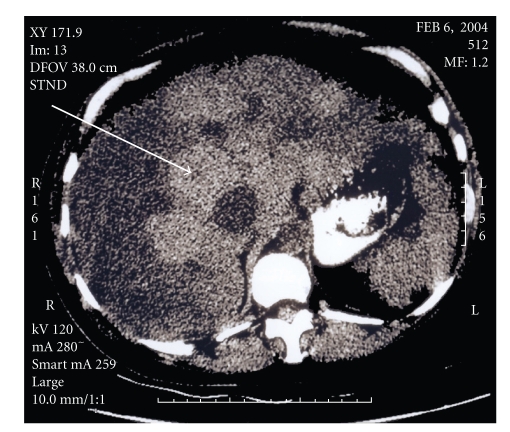
Axial computerized tomography of the abdomen in a liver window demonstrating heterogeneous attenuation of the liver (arrow).
